# Accredited Social Health Activists and pregnancy-related services in Uttarakhand, India

**DOI:** 10.1186/1753-6561-6-S1-P4

**Published:** 2012-01-16

**Authors:** Amit Shukla, Tarun Bhatnagar

**Affiliations:** 1 Department of health and family welfare, Government of Uttarakhand, India; 2 ICMR School of Public Health, National Institute of Epidemiology, Chennai, India

## Introduction

Accredited social health activist (ASHA) is a key link to public health services in villages in India. We conducted a cross-sectional study to determine the proportion of women utilising services of the ASHA for pregnancy-related conditions. We assessed the knowledge, attitude, practices, hindrances and motivation factors among ASHAs regarding pregnancy-related conditions. We also sought to determine the factors associated with the utilisation of ASHAs for pregnancy-related services.

## Methods

Using cluster sampling for the women (18-45 year age group), who delivered in past one year we calculated a sample size of 240 assuming 9% prevalence of ASHA use for antenatal care services with1.9 design effect, 95% confidence interval and cluster size of 10. We selected 24 revenue villages as clusters with probability proportional to number of women in reproductive age group in the villages of Rudraprayag district of Uttarakhand. From each cluster we selected 10 eligible women from consecutive households and all the ASHAs (24) working there.

We used a questionnaire in Hindi (the local language) and administered by trained field workers. We collected data on the socio-demographic and utilisation characteristics of ASHA services for eligible women along with the socio- demographic characteristics and training, knowledge, practices, community acceptance, hindrances and motivational factors for ASHAs.

We used multivariate logistic regression to identify the characteristics of both the eligible women and ASHAs that were independently associated with the utilisation of ASHAs for pregnancy- related services. We calculated the odds ratios and 95% confidence intervals separately for antenatal care (ANC), delivery-related and postnatal care (PNC) services.

## Results

Of the 240 women in the survey, 188 women had heard about ASHA. Of these women who had heard about ASHA 69.7% took the help of the ASHA for antenatal care (ANC), 58.5% took their help for delivery-related conditions and 53.7% took their help for post-natal care (PNC) services. Among the 24 ASHAs in the study, 22 (91%) reported escorting pregnant women for services, 13 (54.2%) had knowledge of being appointed by the panchayat, 19 (79.2%) reported spreading health awareness as one of their job responsibilities, and 15 (62%) reported participating in sit-in protest. 7 (29%) ASHAs did not have community support for their work as ASHA, 18 (75%) ASHAs reported lack of facilities for institutional delivery as hindrance to their work and 12 (50%) reported helping the needy as the major motivation factor for their work.

Utilisation of ASHA’s services was significantly more among women with ASHA as source of *Janani Suraksha Yojana* (a maternal conditional cash transfer scheme) information and motivation for registration, receiving free medicines from ASHA, ASHA with longer work experience and training, and more ASHAs in village. Place of delivery and attending Village Health and Nutrition Day (VHND) were significantly associated with utilisation of ASHA for both delivery and PNC services. Education status of women and ASHA, issues discussed in VHND and distance to health facility were significantly associated with utilisation of ASHA for ANC, delivery and PNC services respectively.

## Discussion

Utilisation of ASHA for ANC was high but lower for delivery-related and PNC services. ASHAs have optimal knowledge of expected work and are the major source of information and support for pregnancy-related services.

